# Linear Motif-Mediated Interactions Have Contributed to the Evolution of Modularity in Complex Protein Interaction Networks

**DOI:** 10.1371/journal.pcbi.1003881

**Published:** 2014-10-09

**Authors:** Inhae Kim, Heetak Lee, Seong Kyu Han, Sanguk Kim

**Affiliations:** 1 Department of Life Sciences, Pohang University of Science and Technology, Pohang, Korea; 2 School of Interdisciplinary Bioscience and Bioengineering, Pohang University of Science and Technology, Pohang, Korea; Institute for Research in Biomedicine, Spain

## Abstract

The modular architecture of protein-protein interaction (PPI) networks is evident in diverse species with a wide range of complexity. However, the molecular components that lead to the evolution of modularity in PPI networks have not been clearly identified. Here, we show that weak domain-linear motif interactions (DLIs) are more likely to connect different biological modules than strong domain-domain interactions (DDIs). This molecular division of labor is essential for the evolution of modularity in the complex PPI networks of diverse eukaryotic species. In particular, DLIs may compensate for the reduction in module boundaries that originate from increased connections between different modules in complex PPI networks. In addition, we show that the identification of biological modules can be greatly improved by including molecular characteristics of protein interactions. Our findings suggest that transient interactions have played a unique role in shaping the architecture and modularity of biological networks over the course of evolution.

## Author Summary

Modular architecture is important for the evolution of cellular systems. Modular rearrangements facilitate functional innovations and modular insulations provide robustness to perturbations. However, molecular-level understanding of the mechanisms underlying modular network evolution is currently not well understood. Here we show that strong domain-domain interactions (DDIs) and weak domain-linear motif interactions (DLIs) made different contributions to the evolution of the modular architecture of PPI networks. Especially, DLIs mediate between-module interactions, and that their relative abundance has dramatically increased in metazoan species. Linear motifs have been identified as evolutionary interaction switches since subtle amino acid changes can cause the short sequences in linear motifs to appear and disappear. Our results suggest that subtle changes in linear motifs have contributed to the rewiring of functional modules and, consequently, to functional innovations in metazoan species.

## Introduction

Biological modules have played an important role in the evolution of cellular systems. After all, it is a group of genes, rather than a single gene, that cooperatively carries out cellular functions and determines phenotypic consequences [1], [2]. Modules facilitate functional innovations in cellular systems, as modular rearrangements provide an efficient way to invent new cellular functions with a limited set of genes [3], [4]. Moreover, modular architecture confers evolutionary robustness and stability to a system, by insulating it from the perturbing effects of genetic variation [5], [6]. However, molecular-level understanding of the mechanisms underlying modular change in complex biological systems is currently not well developed.

Current approaches to identifying modules in protein-protein interaction (PPI) networks often fail to consider the molecular components of connections. Hence, they cannot explain the molecular characteristics underpinning the evolution of network modules. Instead, they often rely on network topology, describing the organization of protein interactions [7]–[9]. Algorithms build topological clusters from protein interactions and try to identify clusters that correspond to certain biological modules, such as functional groups, protein complexes, and subcellular localizations. However, these approaches usually treat all interactions as equal and ignore differences in the nature of the connections.

**Figure 1:**
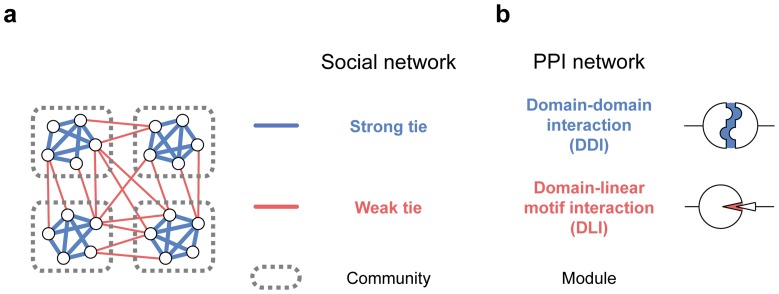
Interaction strength and modular architecture in networks. (**a**) The relationship between tie strength and community structure is well established in social networks. (**b**) DDIs and DLIs correspond to strong and weak interactions in PPI networks, respectively.

Social network studies have shown that network architecture and evolution are closely related to interaction strength [10], [11]. Specifically, strong interactions, or long-term and intense commitments between people, are most likely to exist within communities (Figure 1a). By contrast, weak interactions, or transient and distant acquaintances between people, tend to connect individuals in different communities. This pattern has an evolutionary origin: two unfamiliar people are more likely to develop a social tie and build a community if both of them have strong interactions to a common person [10]. Interaction strengths also influence how global networks function, including the rate and direction of information propagation [11]. Given that biological and social networks often share similar design principles, we anticipated that interaction strength would also affect the evolution of the modular architecture of biological networks.

The physical characteristics of protein interactions are largely determined by their interface structures, which in general are classified into two groups: domain-domain interactions (DDIs) and domain-linear motif interactions (DLIs) [12]. DDIs usually display 10^3^–10^6^ fold stronger affinities than DLIs. Domains are globular structures of long peptides with defined binding or catalytic activities, whereas linear motifs are short peptides composed of specific sequence patterns that bind to other domains. Due to structural differences in the interacting components, DDIs tend to be characterized by large, strong interfaces between two globular domains, whereas DLIs are typically composed of small, weak interfaces between short peptides. In addition, domains and linear motifs have evolved in distinct manners. Domains are often conserved over a wide evolutionary range, evolving in a divergent manner [13], whereas linear motifs tend to emerge from few substitutions in short peptides [14], [15]. Therefore, we hypothesized that DDIs and DLIs may have made different contributions to the evolution of the modular architecture of PPI networks (Figure 1b).

In this study, we investigated the role of DLIs and DDIs in biological modules and found that DLIs are more likely to connect proteins between different biological modules, whereas DDIs tend to connect proteins within the same biological modules, including functional groups, protein complexes, and subcellular localizations. Furthermore, evolutionary analysis of PPI networks revealed that an expansion of DLIs in complex organisms has contributed to an increase in modularity, which may compensate for the cost of network complexity during evolution. We also demonstrated that module identification could be improved by utilizing DLI/DDI information. Indeed, interaction strength represents a unique biological aspect of network modules, one not incorporated by topology information alone. Our study suggests that inclusion of the physical characteristics of protein interactions will improve our understanding of the architecture and evolution of PPI networks.

## Results

### Classifying DDIs and DLIs in the human PPI network

**Figure 2:**
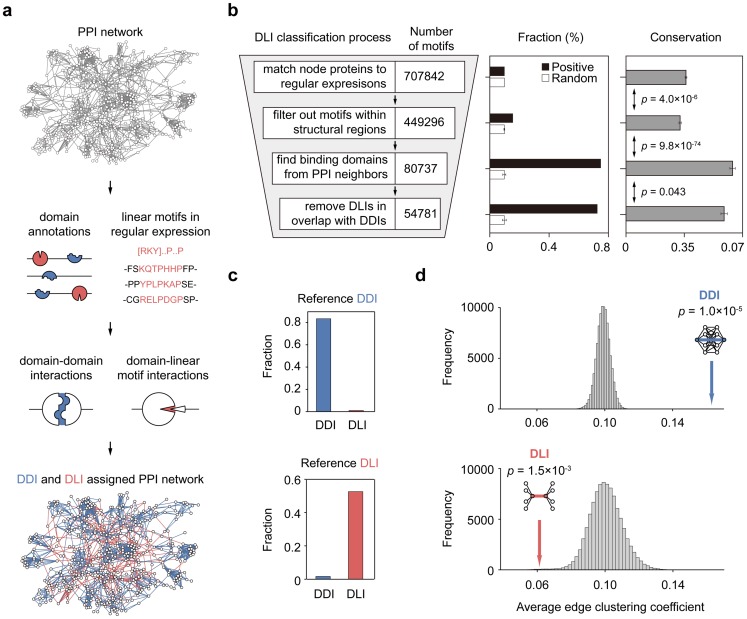
DDI and DLI-assigned human PPI network. (**a**) Categorizing human PPIs as DDIs or DLIs. A part of the human PPI network is shown to visualize DDI/DLI-assigned network. (**b**) Quality assessment of linear motifs during classification process. (**c**) Comparison of DDIs and DLIs categorized using our method to reference sets. (**d**) Edge clustering coefficients of DDIs and DLIs in the human PPI network. Grey bars show the distribution of average edge clustering coefficients in 10^5^ networks with randomly assigned DDIs and DLIs.

We classified human PPIs into DDIs and DLIs to investigate the relationship between interaction strength and the modular architecture of networks (Figure 2a; see Materials and Methods). Briefly, we categorized PPIs as DDIs if two interacting proteins had one or more domain-domain interactions. Interacting domain pairs were either identified directly from 3D structures of protein complexes [12], [16] or from databases of domain-domain pairs [17]. We categorized PPIs as DLIs if two interacting proteins had one or more interacting domain-linear motif pairs. Interacting domain-linear motif pairs were identified from the Eukaryotic Linear Motif (ELM) database, which catalogs sequence patterns of linear motifs using regular expression and their interacting domains [18]. This procedure resulted in an integrated human PPI network containing 39,707 DDIs and 25,093 DLIs (Table S1).

We found that the quality of linear motifs increased during DLI classification steps. Because linear motifs have high rate of false positives [18], we assessed the fraction of true positive motifs in each step of DLI classification. A positive set of 695 experimentally validated motifs were collected from the ELM database and compared with randomly selected ones (see Materials and Methods). We found that the fraction of true positive motifs significantly increased during the classification steps, especially, at the steps exploiting PPI neighbors to detect motif-binding domains and further removing overlap with DDIs (Figure 2b). In contrast, the fraction of random sets remained unchanged during the steps. We also assessed the conservation of motifs since it has been reported that motifs involved in PPIs are relatively conserved [19]. We found that motifs selected from the classification steps are more conserved (Figure 2b). Briefly, conservation score was calculated based on the information entropy of each column in multiple sequence alignments of orthologs and standardized over flanking residues (see Materials and Methods).

We further compared assigned DDIs and DLIs to reference sets in which the interfaces of human PPIs were identified directly from 3D structures or the literature (see Materials and Methods). We found that assigned DDIs and DLIs accorded well with the reference sets (Figure 2c). Specifically, 83.6% of the assigned DDIs (*n* = 816) matched the reference DDIs, whereas only 1.0% of the assigned DLIs (*n* = 10) were included in the reference DDI set. By contrast, 52.6% of the assigned DLIs (*n* = 92) matched the reference DLIs, whereas only 1.7% of the assigned DDIs (*n* = 3) were included in the reference DLI set. This also validates our approach to a classification of PPIs into DDIs and DLIs.

### DDIs and DLIs have different topological roles in the network

We found that DDIs and DLIs have distinct roles in organizing the modular architecture of the human PPI network. DDIs tend to link proteins within the same topological clusters, whereas DLIs are more likely to connect different topological clusters in the network (Figure 2a). To quantify this observation, we investigated the edge clustering coefficients of DDIs and DLIs (see Materials and Methods). The edge clustering coefficient measures the fraction of connections between neighbors of two proteins connected by a given interaction [20]. Thus, interactions with a high clustering coefficient tend to connect proteins within the same topological cluster. We discovered that DDIs have higher edge clustering coefficients than DLIs (Figure 2d, colored arrows). The average clustering coefficient of DDIs was 0.16 and that of DLIs was 0.061 (Kolmogorov-Smirnov test, *p* = 1.0×10^−323^).

We confirmed that the observed clustering coefficients of DDIs and DLIs could not occur by random chance comparing them to randomly assigned ones (Figure 2d, grey bars). The randomly assigned DDIs and DLIs were constructed by shuffling domains and linear motifs across proteins, while keeping the network connections unchanged (see Materials and Methods). Note that false classification of DDIs or DLIs would lead the clustering coefficient similar to that of random ones because the network topology was not changed. The high clustering coefficients of actual DDIs and the low clustering coefficients of actual DLIs were significantly different than those of randomly assigned ones (*p* = 1.0×10^−5^ for DDIs; *p* = 1.5×10^−3^ for DLIs). This was further confirmed based on the conservation of motifs constituting DLIs. We changed DLI datasets by varying motif conservation scores and measured average clustering coefficients. We found that the average clustering coefficients of DLIs were lower than that of DDIs, regardless of their motif conservation scores (Figure S1). Interestingly, the average clustering coefficients even decreased as the conservation of motifs increased. These indicate that the observed clustering coefficient would not likely emerge from false classifications.

Because of the degeneracy in regular expressions, certain motifs could stochastically occur in many proteins. Therefore, we removed DLIs with low information content and reanalyzed the dataset. We confirmed that clustering coefficients of DLIs were lower than that of DDIs when we removed motifs with higher probability to be found by chance. DLIs showed lower clustering coefficient compared to DDIs even after removed 89 motifs with probability over 10^−5^ (Figure S2a). Moreover, we found that the probability and clustering coefficient of motifs did not show significant correlation (Figure S2b; *p* = 0.15, Pearson's correlation). This confirms that DLIs generally have lower clustering coefficient, which is not restricted to several prevalent motifs.

### DLIs connect different biological modules, while DDIs connect proteins within biological modules

We next compared the role of DLIs and DDIs in various biological modules. Because biological modules are groups of proteins with tight functional relationships [1], we investigated functional groups identified based on Gene Ontology (GO) terms. Protein complexes and subcellular localizations were also investigated, since they represent protein groups with particular functions [21]–[23].

**Figure 3:**
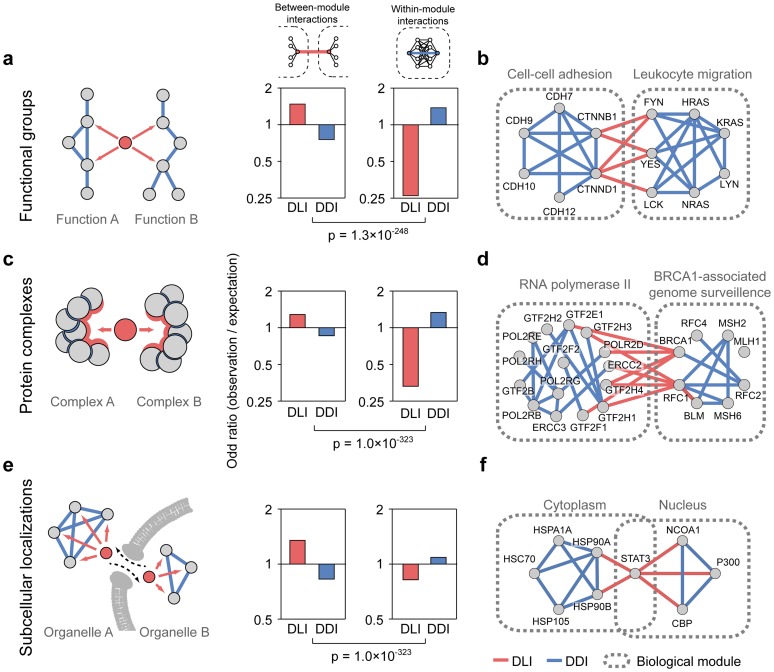
Enrichment of DLIs and DDIs in interactions between and within biological modules. (**a**) Odd ratio in functional groups. (**b**) Two functional groups, ‘cell-cell adhesion’ and ‘leukocyte migration’ were shown. (**c**) Odd ratio in protein complexes. (**d**) Two protein complexes, ‘RNA polymerase II’ and ‘BRCA1-associated genome surveillance’ were shown. (**e**) Odd ratio in subcellular localizations. (**f**) Two subcellular localizations, ‘cytoplasm’ and ‘nucleus’, were shown.

We found that DLIs were enriched in protein interactions connecting different functional groups, whereas DDIs were enriched in interactions connecting proteins within the same functional group (Figure 3a, Table S2). Functional groups were identified using molecular functions (MFs) and biological processes (BPs) based on GO terms, while controlling for module size and overlapping relationships (see Materials and Methods). For example, DLIs mediated by SH2 domains of Src kinase family proteins (FYN, YES, LCK) connect ‘cell-cell adhesion’ and ‘leukocyte migration’ protein groups (Figure 3b). The Src kinases transiently dissociate p120-catenin (CTNND) and cadherins (CDHs) via phosphorylation, which results in short-lived gaps between vascular epithelial cells [24]. This enables leukocytes to transmigrate from blood vessel to tissue, which suggests that DLIs contribute to transient interactions between different functional groups. By contrast, DDIs connect proteins within the ‘cell-cell adhesion’ group through their Arm and Cadherin_C domains. And the proteins within the ‘leukocyte migration’ group are connected by the DDIs of the Pkinase_Tyr and Ras domains. We also confirmed that the bias of DLIs towards between-module interactions was observed regardless of their motif conservation (Table S3).

We found that DLIs were enriched in between-complex interactions, whereas DDIs were enriched in within-complex interactions (Figure 3c, Table S2). For example, DLIs mediated by the BRCT domains of the BRCA1 protein connected the ‘RNA polymerase II’ and ‘BRCA1-associated genome surveillance’ complexes (Figure 3d). The BRCT domain is a phosphopeptide-binding domain that mediates signal transduction events in the DNA damage response pathway [25]. BRCA1 interacts with the phosphorylated and functionally processive form of the RNA polymerase II complex to respond to DNA damage [26], suggesting that DLIs contribute to transient interactions between different protein complexes. By contrast, DDIs connect proteins within the ‘RNA polymerase II’ complex via the TFIIE_alpha and BSD domains. In addition, the proteins within the ‘BRCA1-associated genome surveillance’ complex are connected by DDIs between the MutS and Helicase_C domains.

We found that DLIs were enriched in protein interactions across different subcellular localizations, whereas DDIs were enriched in protein interactions within subcellular localizations (Figure 3e, Table S2). For example, the signal transducer and activator of transcription 3 (STAT3) protein interacts with its partners in the cytoplasm and nucleus via DLIs (Figure 3f). Specifically, the STAT3 protein transiently binds to heat shock protein 90 (HSP90) in the cytoplasm and translocates to the nucleus, where it releases HSP90 to interact with other transcription factors [27]. By contrast, DDIs connect proteins with the same subcellular localization. For example, the Hsp70 and Hsp90 domains participate in protein interactions in the cytoplasm, whereas the Creb binding and Bromo domains participate in those in the nucleus. This suggests that DLIs contribute to the transient interactions of proteins that translocate between different subcellular localizations. We also provide more examples for the enrichment of DLIs and DDIs in interactions between and within biological modules (Figure S3).

We confirmed that DDIs are biased toward within-module interactions regardless of they are mediated by same or different domains. One might ask that the observed bias of DDIs toward within-module interactions emerged from similar functions of identical domains. To test this question, we divided DDIs into two groups, homo- or hetero-DDIs. Any DDIs mediated by one or more pairs of same domains were classified as homo-DDIs and the rest of them were classified as hetero-DDIs based on their Pfam ID. We found that both homo- and hetero-DDIs are biased toward within-module interactions for functional groups, protein complexes, and subcellular localizations (Table S4). This indicates that the observed bias is likely due to the differences between DDI and DLI.

### Metazoan PPI networks: An increase in DLIs accompanies the evolution of modularity

**Figure 4:**
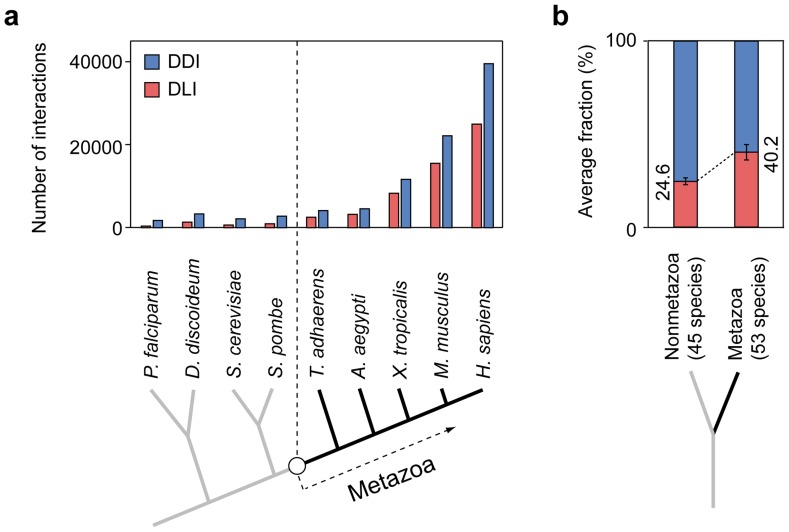
Expansion of DLIs in metazoan PPI networks. (**a**) The number of conserved DLIs and DDIs in eukaryotic species. Values for nine representative eukaryotic species are shown. (**b**) Average proportion of DLIs and DDIs in 45 nonmetazoan and 54 metazoan species.

Next, we investigated how the evolution of DLIs and DDIs contributed to the modular architecture of PPI networks. Comparative genomic studies have revealed that the number of peptide-binding domains and linear motifs, the basic components of DLIs, expanded as the complexity of organism increased [28]. We found the number of DLIs increased sharply in metazoan species (Figure 4a; Table S5). PPI networks for 45 nonmetazoan and 53 metazoan species were constructed using orthologous protein interactions from the human PPI network (see Materials and Methods). Although the number of both DDIs and DLIs increased in metazoan PPI networks, the increase in DLIs was greater than that in DDIs. The average proportion of DLIs was 24.6% in nonmetazoan species; it increased to 40.2% in metazoan species (Figure 4b; *t*-test, *p* = 2.4×10^−43^). As expected, we found that the increases of linear motifs and DLI domains are more significant than that of DDI domains (Figure S4).

**Figure 5:**
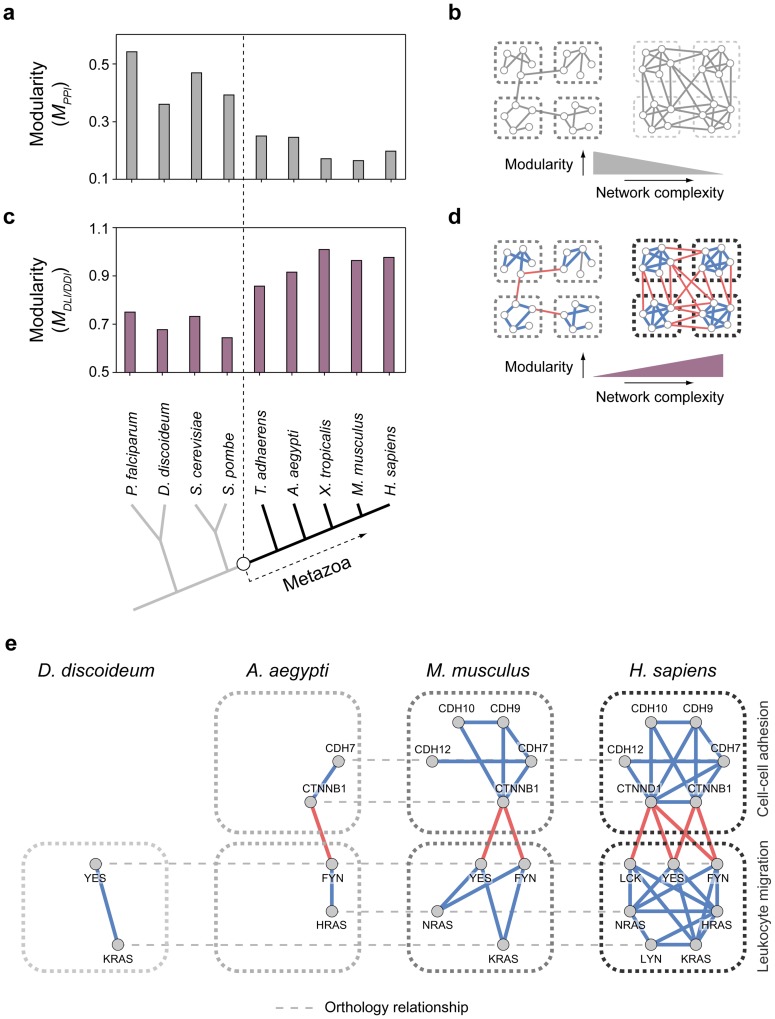
The expansion of DLIs contributed to the increase in modularity of metazoan PPI networks. (**a**) Topological modularity, *M_PPI_*, in nine representative eukaryotic species. (**b**) A schematic showing how increased complexity is associated with *M_PPI_*. (**c**) Network modularity (*M_DLI/DDI_*) in nine representative eukaryotic species. (**d**) A schematic showing how DLIs are associated with increased *M_DLI/DDI_*. (**e**) The evolution of ‘cell-cell adhesion’ and ‘leukocyte migration’ groups is shown as an example.

What was the impact of this increased proportion of DLIs upon metazoan PPI networks? We measured the modularity of PPI networks in eukaryotic species and found that the expansion of DLIs contributed to the modular architecture of metazoan PPI networks. To quantify the modularity of PPI networks in different species, we first applied a widely accepted topological measure, *M_PPI_*. By measuring the enrichment of within-module interactions, this measure was designed to assess to what extent modules are separated from each other (see Materials and Methods). We discovered that the *M_PPI_* decreased sharply in metazoan PPI networks relative to those of nonmetazoans (Figure 5a, Figure S5). This decreased *M_PPI_* was due to an increase in between-module interactions, which connect proteins in different modules and reduce module boundaries (Figure 5b, Table S5). For example, the fraction of between-module interactions for protein complexes was 45.3% in nonmetazoans and 65.3% in metazoans (Figure S6; *p* = 2.0×10^−24^). We again tested whether the decrease of *M_PPI_* is due to any evolutionary association from same domains and found that *M_PPI_* decreased for both homo- and hetero-DDIs (Figure S7, S8).

Connections between different modules, however, do not necessarily reduce the modularity of PPI networks, because transient interactions between different modules are critical to the proper function of modular architecture. Therefore, we formulated a new modularity measure, *M_DLI/DDI_*, which takes into account DLI/DDI information; it incorporates the idea that DLIs mediate interactions between different modules, whereas DDIs mediate interactions within the same modules (see Materials and Methods). In contrast to the decrease observed in the *M_PPI_*, we discovered that the *M_DLI/DDI_* increased in metazoan PPI networks relative to nonmetazoan networks (Figure 5c, Figure S5, S7, S8). Indeed, we found that DLIs tend to connect proteins at module boundaries, improving module quality in complex PPI networks (Figure 5d). For example, novel Src family kinase (FYN, YES, LCK) DLIs emerged in metazoan species, regulating the transient opening of the junction between vascular epithelial cells in leukocyte migration [24]. Because of abundant connections between the two modular groups, each module's boundary is unclear at first glance. However, DLIs mediate the between-module connections of leukocyte migration and cell-cell adhesion modules, helping them cluster independently (Figure 5e).

### DLI/DDI information improves identification of biological modules in PPI networks

**Figure 6:**
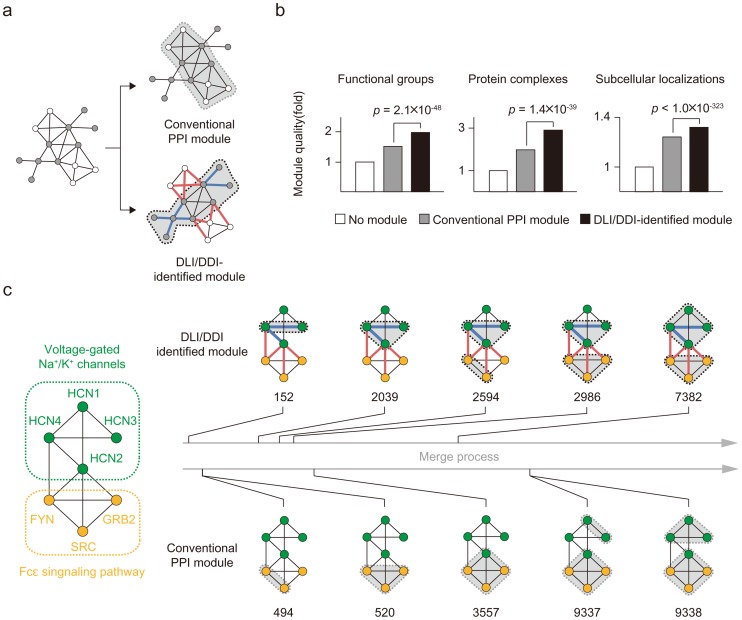
Employing DLI/DDI information to identify biological modules. (**a**) DLI/DDI information improves the identification of biological modules. (**b**) Quality of modules identified using conventional PPI data vs. DLI/DDI data. Module quality reflects the similarity of biological annotations in protein pairs within modules. (**c**) A detail of the merge process for conventional PPI and DLI/DDI-identified modules. The two horizontal arrows represent the merge process for seven proteins associated with ‘Voltage-gated Na^+^/K^+^ channels’ and ‘Fcε signaling pathway’. Ordinal numbers of specific merge steps are shown.

Because DLIs and DDIs have distinct roles in the modular architecture of PPI networks, we employed DLI/DDI information in a topology-dependent module detection algorithm to improve identification of biological modules. We anticipated that DDIs would cluster proteins into modules, since they connect proteins with the same biological functions, whereas DLIs would separate proteins into different modules, since they involve transient interactions between proteins with different biological functions (Figure 6a). To test this idea, we compared conventional topological PPI modules and DLI/DDI-identified modules. We constructed conventional PPI modules by using a greedy module-optimization algorithm, which consecutively merged single nodes to determine the architecture with the highest modularity (see Materials and Methods). To construct improved modules, we applied DLI/DDI information by adjusting interaction weights.

We found that considering DLI/DDI information dramatically improved the identification of biological modules (Figure 6b). The quality of DLI/DDI-identified modules was significantly better than that of conventional PPI modules; this was true of various biological modules, including functional groups, protein complexes, and subcellular localizations. To quantify module quality, we analyzed the similarity of functional annotations, membership in protein complexes, and localization of subcellular compartments (see Materials and Methods). The quality of functional groups was analyzed in terms of both MF and BP terms. We found that DLI/DDI-identified modules showed better quality than conventional PPI modules for various module sizes (Figure S9).

Next, we investigated how DLI/DDI information could improve the merge process, resulting in better-quality protein clusters. By weighting network connections differently, the process prioritized the merging of DDIs in early steps and delayed DLI merges until later steps. For example, we found that voltage-gated Na^+^/K^+^ channel proteins (HCN1-4) were grouped into the same module (Figure 6c). A DDI between HCN2 and HCN4 ensured the merging of the two proteins in an early step. Conversely, DLIs between HCN proteins and Fcε signaling proteins (FYN, SRC, GRB2) delayed the merge events for these proteins, resulting in separate modules. By contrast, based on conventional PPI information alone, HCN2 clustered with the FYN, SRC, and GRB2 proteins, becoming a member of the same functional module. This indicates that DLI/DDI information can improve the functional annotation process by identifying biologically relevant modules not easily identified using network topology alone.

## Discussion

In this study, we show that interaction strength plays a crucial role in shaping biological modules. Specifically, weak and transient interactions between modules promote the formation of functionally competent modular architecture in PPI networks, while a growing number of proteins and interactions have increased network complexity. Interestingly, it has been reported previously that weak interactions are enriched in between-module connections and are important for the proper function of various complex networks. For example, in social networks, weak interactions across community boundaries serve as passages along which novel information can travel [10]. Similarly, in the human brain, weak interactions connecting functional modules maximize information transfer at minimal wiring cost [29]. Indeed, interactions mediated by linear motifs are enriched in signaling and post-translational regulation networks [30], [31]. This suggests that transient interactions mediating connections between modules may be a common design principle in complex networks. Thus, we propose that incorporating interaction strength into the study of network architecture provides novel insight into the principles of organization in biological systems.

Due to the unstable characteristics, transient interactions are more difficult to detect than stable interactions [31]. We tested whether our conclusion is robust to underestimated transient interactions. Because multiple reports likely indicate more stable PPIs [32], we constructed a stable PPI (SPPI) network using the PPIs found from two or more source of publications. We found that the clustering coefficient of DLIs was significantly smaller than that of DDIs (Figure S10; *p* = 3.7×10^−53^, *u*-test). We also found that DDIs and DLIs in SPPI network are enriched in within- and between-module interactions, respectively (Table S6). Therefore, we expect that our conclusions remain unchanged against future expansion of PPI networks with more transient interactions.

We showed that DLI/DDI information can improve the identification of biological modules (Figure 6). Here, we focused on finding modules based on a conservative way, in which modules likely comprise strong DDIs between proteins with similar functions. Therefore, DLIs had been weighed lower than DDIs using a conventional framework which was designed to separate topological clusters. However, one might have another motivation of finding dynamic modules composed of transient interactions. We expect that DLIs and DDIs would also be informative in such cases because transient PPIs involved in dynamic cellular functions are likely mediated by DLIs [30], [31]. One immediate way of finding dynamic modules would be to weigh DLIs over DDIs to find modules comprising DLIs rather than DDIs. This idea could be systematically tested when there were more experimental evidences for dynamic modules available from the advancement of detection methods for transient interactions [33], [34].

We found that complex PPI networks displayed highly modular architecture when transient interactions were taken into account. Without proper consideration of transient interactions, however, complex PPI networks appeared to have lower levels of modularity than simple ones (Figure 5). It has been suggested that modular architecture is crucial in highly complex biological systems, to alleviate the “cost of complexity” during evolution [35]. For example, modules confer robustness to biological systems by insulating against the spread of perturbations originating from genetic variation. Without such insulation, perturbations could alter various functions, which would be likely to result in undesirable changes. Insulation becomes more critical as the complexity of biological systems increases; complex networks contain more components that can be perturbed than do simple ones [36]. In general, yeast and mouse experiments have shown that the effect of a single mutation is restricted, affecting a few traits [5], [6]. This implies that modular pleiotropic structure does exist in the genotype-phenotype relationship. Our results highlight the fact that transient interactions are key in shaping the modular architecture of complex PPI networks.

We found that DLIs mediate between-module interactions and that their relative abundance has dramatically increased in metazoan species. Functional innovations in metazoan species have often emerged from the rewiring of conserved functional modules [3], [37], [38]. Therefore, DLIs may be a key component of the rewiring of different functional modules in PPI networks. Indeed, linear motifs have been identified as “evolutionary interaction switches,” because subtle amino acid changes can cause the short sequences in linear motifs to appear and disappear [14], [15], [39]–[41]. Furthermore, structurally disordered regions, where linear motifs are often located, have a high capacity for evolutionary rewiring in PPI networks [42] and largely increased in complex organisms [43]. This “switch-like” characteristic of short sequence motifs has been regarded as a prominent evolutionary mechanism affecting developmental processes in metazoan species. For example, mutations in *cis*-regulatory elements can selectively alter the expression of specific functional modules and result in dramatic changes in morphological patterns [44], [45]. Our results suggest that subtle changes in short coding region peptides have also contributed to the rewiring of functional modules and, consequently, to functional innovations in metazoan species.

## Materials and Methods

### Integrated human PPI networks

To assign DDI and DLI status, we first collected human PPI data from the following databases: the Human Protein Reference Database (HPRD), release 9 [46]; BioGRID, release 3.2.107 [47]; IntAct [48], downloaded December 3, 2013; the Molecular Interaction Database (MINT) [49], released March 26, 2013; the Database of Interacting Proteins (DIP) [50], released October 29, 2013; Reactome v46 [51]; MatrixDB [52], released August 1, 2012; and InnatedDB [53], released July 11, 2013. The integrated human PPI network comprised 264,845 interactions between 15,857 proteins.

### Classification of DDIs

We classified a PPI as a DDI if two partner proteins had one or more interacting domain-domain pairs. Data on human protein domains were obtained from the Protein Family Database (Pfam), release 27.0 [13]. Interacting domain-domain pairs were either identified directly from 3D structures or predicted using various computational approaches [17]. We first obtained 9,616 structurally characterized interacting domain-domain pairs from the Database of Three-dimensional Interacting Domains (3did), downloaded October 31, 2013 [12] and iPfam, release 1.0 [16], regarding them as the gold standard set. Then, every predicted interaction between domain-domain pairs received a confidence score:
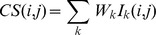
where *CS*(*i*,*j*) is the confidence score for the pair domain *i* and domain *j*, *k* indicates the prediction method, *W* is a precalculated weight factor for a specific prediction method, and *I* is an indicator of the prediction result (*I_k_*(*i*,*j*) = 1 if the method *k* gives a positive prediction for the pair domain *i* and domain *j*; *I_k_*(*i*,*j*) = 0 otherwise). The weight factor assigned each prediction method was equal to its precision:
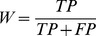
where TP is the number of true positives, or the number of domain-domain pairs predicted by a given method and found in the gold standard set, and FP is the number of false positives, or the number of domain-domain pairs predicted by a given method but missing from the gold standard set. Predicted interactions between domain-domain pairs were considered valid if their confidence scores were greater than a cutoff value (*CS_0_*). To select a reliable *CS_0_*, we investigated the *F_1_* score of prediction results, increasing *CS_0_* from 0 to 1.20 in 0.01 increments (Figure S11). The *F_1_* score is the harmonic mean of precision and recall:
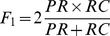
where PR and RC are the precision and recall, respectively, of predicted interactions between domain-domain pairs with a *CS*>*CS_0_*. Precision and recall were calculated as follows:
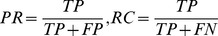
where TP is the number of domain-domain pairs with *CS*>*CS_0_* that were present in the gold standard set; FP is the number of domain-domain pairs with *CS*>*CS_0_* that were missing in the gold standard set; and FN is the number of domain-domain pairs with *CS*<*CS_0_* that were present in the gold standard set. Using the *CS_0_* with the greatest *F_1_* (*CS_0_* = 0.13, *F_1_* = 0.128), we obtained 6,911 interacting domain-domain pairs predicted using various computational approaches. In total, this procedure gave us 16,527 interacting domain-domain pairs from both 3D structures and predictions. To avoid any bias in biological modules, we excluded prediction methods that exploited functional similarity.

### Classification of DLIs

We classified a PPI as a DLI if two partner proteins had one or more interacting domain-linear motif pairs. We identified linear motifs in human proteins using regular expressions that represent motifs [18]. In contrast to other approaches, regular expressions have the flexibility to account for short indels and to provide presence/absence matches for motif patterns, simplifying the search. This feature is pertinent to our method, because interactions at the protein level will filter out most over-determined motifs. Two context filters provided by ELM server were also applied to the search. A taxonomic range filter removed linear motifs not related to human sequences. A structure filter removed linear motifs that overlapped with predicted secondary structures in globular domains. Interacting domain-linear motif pairs were obtained from “ELM classes” [18]. Each ELM class represents a pair of motif patterns and domains that interact with each other. Among the six types of ELM classes, we used ligand binding sites (LIG), docking motifs (DOC), and degron motifs (DEG) to focus on protein binding rather than the cleavage, targeting, or modification of motifs. PPIs remained unclassified if they satisfied criteria for both DDIs and DLIs. In total, we assigned 39,707 DDIs and 25,093 DLIs to 9,585 proteins.

### Quality assessment of linear motifs

ELM instances, experimentally validated motifs in ELM database, were downloaded June 12, 2014. Among them, we found 695 positive and 12 negative motifs presented in the network. Because the number of negative motifs were too small to assess quantitatively, we also generated 10,000 random sets comprising 695 motifs of random selection for each and compared them to the positive set.

We assessed the conservation of a motif using relative local conservation score (RLC) for each comprised residue and took their average for the motif [54]. RLC was calculated as follows:
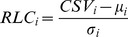
where CSV means conservation of residues from information entropy, *μ_i_* and *σ_i_* are mean and standard deviation of CSV, respectively, of [*i*−10,*i*+10] residues including residue *i* itself. We used Shannon's entropy of each column in aligned ortholog sequences as CSV:
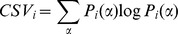
where *i* denotes each column, *α* is an amino acid presented in a column, and *P*(*α*) is the frequency of the amino acid *α* in a column. Orthologs were obtained from Inparanoid database and only 100% confidence orthologs were used [55]. Otholog sequenes were aligned by MUSCLE algorithm [56]. For Figure S1 and Table S3, DLIs were ordered by the highest conservation of comprising motifs and divided into different groups.

### Reference sets of DDIs and DLIs

We collected reference sets of human DDIs and DLIs whose status could be directly ascertained from 3D structures and literatures. Although 3did, iPfam and ELM databases provided experimentally confirmed DDIs and DLIs, only part of them might be interactions found in human proteins. Therefore, we chose reference DDIs from 3did and iPfam, if two protein constructs in the experiment were derived from human sequences by tracking species information from Protein Data Bank [57]. Reference DLIs were collected from ELM interactions by filtering out species other than human. Overlaps between reference DDIs and DLIs were discarded. The procedure resulted in 976 reference DDIs and 175 reference DLIs.

### Topology difference between DDI and DLI

Edge clustering coefficient measures the ratio of observed cyclic structures over possible cyclic structures around two connected nodes. Specifically edge clustering coefficient, *C*, between two nodes, *i* and *j*, was measured as follows [20]:
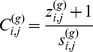
where 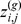 is the number of observed cyclic structures and 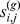 is the number of possible cyclic structures among the partners of node *i* and *j*; *g* is the order of cycles, i.e. the number of nodes included in each cyclic structure. Here, we set *g* = 4. We generated 10,000 permutations of DDIs and DLIs to obtain empirical *p*-values for the clustering coefficients. We permuted domains and linear motifs preserving their number in each protein and reassigned DDIs and DLIs.

### Establishing biological modules

By definition, biological modules in PPI networks are groups of proteins that have tight functional relationships [1]. To determine functional groups of proteins, we used GO annotations, which provide a wide range of descriptions for the cellular function of proteins [58]. However, GO terms do not directly facilitate a clear division among functional groups, as they are designed to create hierarchical relationships in which parent terms include their child terms. To employ GO terms in a way that clearly separated functional groups, we first gathered certain GO terms with a comparable number of annotated proteins. We removed GO terms that displayed high levels of overlap, excluding the smaller of two GO terms when the union of the pair contained more than 50% of associated proteins. The procedure described was performed on terms from two functional GO categories: MF and BP.

For protein complexes, we used the Mammalian Protein Complexes (CORUM) database [59]. We employed only human complexes, to prevent any bias originating from the higher level of conservation observed in DDIs [39]. Since several protein complexes with little variation can emerge from a subtle difference in the conditions employed in detection experiments, we removed those with high levels of overlap. As for functional groups, we excluded the smaller of two complexes whose union shared more than 50% of associated proteins. This procedure resulted in 1,217 protein complexes comprised of 2,646 proteins.

We used the consensus localization prediction (ConLoc) method [22] to analyze subcellular localization. The algorithm first uses Universal Protein Resource (Uniprot) annotations, if available [60]. Then, it gives multiple predictions for subcellular localizations of a given protein, including associated confidence levels. In the cases in which no Uniprot annotation was available, we used the best prediction as the localization; we included the second prediction as well, if it was assigned over 80% confidence. This procedure resulted in 9 subcellular localizations for 18,575 proteins.

### Enrichment of DLIs and DDIs in between and within-module interactions

To investigate the role of DLIs and DDIs in biological modules, we classified PPIs as within-module or between-module interactions. PPIs were considered within-module interactions if the interacting proteins had identical module memberships. Conversely, PPIs were considered between-module interactions if the interacting proteins had no common module membership. However, there were PPIs that met neither of these criteria (dubbed “overlapping interactions” in Figure S12). These overlapping interactions connected proteins that shared only part of their module memberships; thus, they could be interpreted either as within-module or between-module interactions. To be robust, we built two datasets. One treated overlapping interactions as within-module interactions, and the other classified overlapping interactions as between-module interactions. In both sets, our results were qualitatively similar, demonstrating that DLIs were enriched in between-module interactions and DDIs were enriched in within-module interactions (Table S2).

Next, we further characterized the association of DLIs and DDIs with between and within-module interactions. We constructed a 2×2 contingency table with four types of interactions: between-module DLIs (*n*_11_), between-module DDIs (*n*_12_), within-module DLIs (*n*_21_), and within-module DDIs (*n*_22_). Enrichment was calculated as the observed number of interactions over the expected number of interactions for a specific association. For the observed number of *n_xy_*, the expected number was calculated as 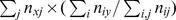. For example, the expected number of between-module DLIs was (*n*_11_+*n*_12_)×(*n*_11_+*n*_21_)/(*n*_11_+*n*_12_+*n*_21_+*n*_22_), i.e., the number of between-module interactions multiplied by the fraction of DLIs among the annotated proteins. We also determined if the level of enrichment was significant by calculating the *p*-value from Fisher's exact test. An analysis of MF terms for modules sized 80–160 proteins is shown in Figure 3.

### PPI networks for eukaryotic species

We used protein orthology between human and other species to construct PPI networks and their modular architecture, as most interactomes were unknown when the genomes were sequenced. A human PPI was regarded as conserved in other species if the interacting pair of proteins had orthologs in them. Ortholog data were obtained from the Inparanoid database, and only 100% confidence orthologs were used [55]. Ortholog with the longest sequence was chosen, in case of multiple orthologs presented. To assign DDIs and DLIs, we searched domains and linear motifs in each species. To find domains, ortholog sequences were searched against the profile hidden Markov models of Pfam-A domains using pfam_scan.pl script and HMMER3 [13], [61]. Linear motifs were searched using regular expressions and those overlapping with any domain region were discarded [18]. In this way, we constructed PPI networks for 45 nonmetazoan and 53 metazoan species.

### Measuring modularity

We used Newman modularity to measure *M_PPI_* [9]. The key assumption underlying topological modularity is that modules are separated from each other; the nodes within each module are densely connected, and the nodes between modules are sparsely connected. Specifically, topological modularity was calculated as follows:
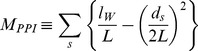
where *l_W_* is the number of interactions that connect proteins within the module, *L* is the number of interactions in the network, and *d_S_* is the sum of node degrees in the module. It measures the extent to which the proportion of observed within-module interactions exceeds the proportion expected by chance.

However, *M_PPI_* strictly focuses on the separation of modules in network architecture, failing to recognize that biological modules influence each other. Indeed, the best *M_PPI_* score occurs when biological modules have no connection, which is unnatural. Given that DLIs likely connect different biological modules to carry out cellular functions, we revised *M_PPI_* to reflect that DDIs contribute to within-module interactions and DLIs contribute to between-module interactions. The revised modularity value, *M_DLI/DDI_*, was calculated as follows:
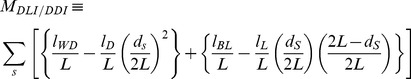
where *l_WD_* is the number of DDIs that connect proteins within the module, *l_D_* is the number of DDIs in the network, *l_BL_* is the number of DLIs that connect proteins in the module to proteins outside the module, and *l_L_* is the number of DLIs in the network. The proportion expected by chance was adjusted for the proportion of DDIs and DLIs in the network. An analysis of BP terms for modules sized 80–160 proteins is shown in Figure 5.

### Employing DLI/DDI information in module identification

To identify conventional PPI modules, we used a greedy modularity optimization algorithm [62]. Initially, each node was treated as a single module. Then, the algorithm merged nodes consecutively, until the entire network became a single module. In each step, all possible merge events between interacting nodes were evaluated by calculating changes in topological modularity, and the merge event with the greatest (or least decreased) value was selected. Modules were finalized according to the merged group of nodes with the highest modularity. Modules that possessed only two proteins were excluded from the analysis.

We identified DLI/DDI-informed modules based on a procedure similar to the one used to identify conventional PPI modules; however, it weighted DDIs and DLIs differently [63]. In general, PPIs were categorized in a binary manner (1 if they existed, 0 if they did not). When an interaction was assigned to be DDI, its contribution to merging process is greater than a conventional PPI. By contrast, an interaction was assigned to be DLI, its contribution to merging process works in the opposite way. Thus, we weighted DDIs at 100 and DLIs at 0.1. We used community_fastgreedy() function in python-igraph package to build both PPI modules and DLI/DDI-identified modules (http://igraph.org/python/). The resulting modules were provided in Table S7.

### Module quality measure

We assessed module quality by measuring how similar proteins within the same module were. The similarity of each protein pair was calculated as the Jaccard index of biological annotations:
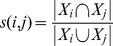
where *i*, *j* is the protein pair and X is the set of biological annotations. Module quality was calculated as the average similarity of protein pairs. Fold increase in module quality was measured by comparing module quality to the average similarity of all protein pairs in the network. The *p*-value comparing module quality between the DLI/DDI-identified modules and conventional PPI modules was calculated using the Kolmogorov-Smirnov test. We also investigated the effect size of employing DLI/DDI information upon module quality using Cohen's *d*, designated *e* in Figure S8. An analysis of MF terms for modules sized 80–160 proteins is shown in Figure 6.

## Supporting Information

Figure S1 Edge clustering coefficient of DLIs with various conservation scores. DLIs were ordered by the conservation of comprising motifs and divided into 10 groups. Numbers in parentheses show the number of DLIs in each group.
(TIF)

Figure S2 Clustering coefficients of DLIs according to the probability of motif regular expression. (a) Clustering coefficients of DLIs with varying probability cutoffs were compared with that of DDIs. (b) Each dot represents a motif with unique regular expression having its probability to be found by chance on x-axis, while average clustering coefficient of interactions on y-axis. The probability of motif regular expression was calculated as the product of the amino acid probability in each position of a motif [18].
(TIF)

Figure S3 Examples of DLIs and DDIs in interactions between and within biological modules for (a) functional groups, (b) protein complexes, and (c) subcellular localizations.
(TIF)

Figure S4 Number of domains and linear motifs mediating DDIs and DLIs in the PPI networks of eukaryotes. Average number per protein was calculated for each species and *p*-value was taken comparing 45 nonmetazoan and 53 metazoan species by *t*-test.
(TIF)

Figure S5 Enrichment of DLIs and DDIs in between and within-module interactions. Non-overlapping GO terms were used as functional groups. Module size represents the number of proteins in a given GO term.
(TIF)

Figure S6 Enrichment homo-DDIs and DLIs in between and within-module interactions.
(TIF)

Figure S7 Enrichment hetero-DDIs and DLIs in between and within-module interactions.
(TIF)

Figure S8 Increase in between-module interactions in metazoan PPI networks among protein complexes. (**a**) The number of between and within-module interactions in eukaryotic species among protein complexes. Values are shown for nine representative eukaryotic species. (**b**) Average proportion of between and within-module interactions in nonmetazoan and metazoan species.
(TIF)

Figure S9 Comparison of module quality between DLI/DDI-identified modules and conventional PPI-identified modules for different functional groups. For both molecular function and biological process, the effect size, *e*, and the *p*-value are shown, stratified by module size.
(TIF)

Figure S10 Edge clustering coefficient of DDIs and DLIs in SPPI network.
(TIF)

Figure S11 *F_1_* score for the prediction of interacting domain-domain pairs in relation to the cut-off value, *CS_0_*. The *F_1_* score was calculated for positive predictions, which were domain-domain pairs with a confidence score, *CS*, greater than the *CS_0_*.
(TIF)

Figure S12 Schematic illustrating how PPIs were categorized as between or within-module interactions.
(TIF)

Table S1 DDI and DLI-assigned human PPI network.
(XLSX)

Table S2 Number of DDIs and DLIs as within- or between-module interactions.
(XLSX)

Table S3 Fraction of DLIs with different conservation in between- or within-module interactions.
(XLSX)

Table S4 Enrichment of homo- and hetero-DDIs in interactions between and within biological modules.
(XLSX)

Table S5 Number of interactions in orthologous PPI networks of eukaryotic species.
(XLSX)

Table S6 Number of DDIs and DLIs as within- or between-module interactions in SPPI network.
(XLSX)

Table S7 Module membership of proteins for PPI modules and DLI/DDI modules.
(XLSX)
