# Supplementary figures and images for: Linear Motif-Mediated Interactions Have Contributed to the Evolution of Modularity in Complex Protein Interaction Networks
Source: PLoS Comput Biol. 2014 Oct 9;10(10):e1003881. doi: 10.1371/journal.pcbi.1003881 (PMC4191887; doi:10.1371/journal.pcbi.1003881)

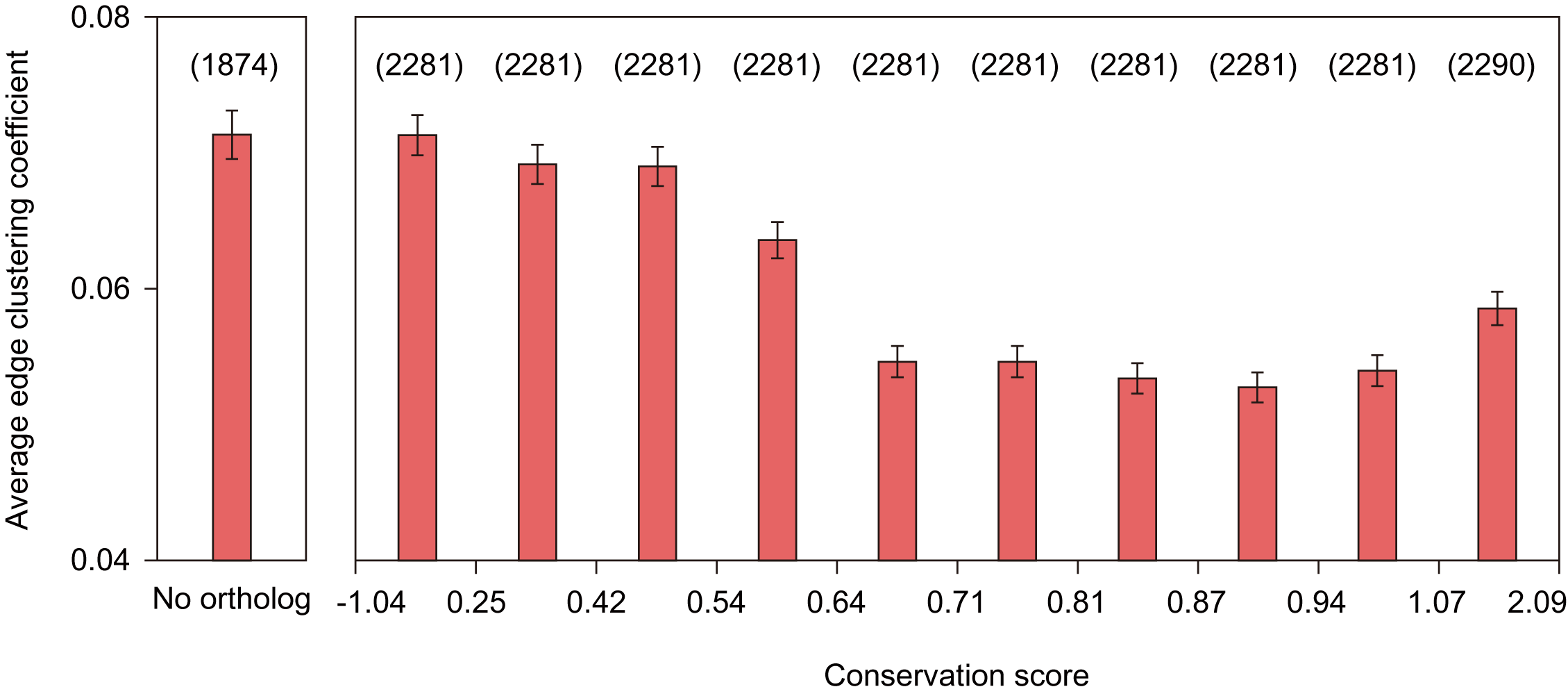

Supplement: Figure S1 — Edge clustering coefficient of DLIs with various conservation scores. DLIs were ordered by the conservation of comprising motifs and divided into 10 groups. Numbers in parentheses show the number of DLIs in each group. (TIF) [file pcbi.1003881.s001.tif]

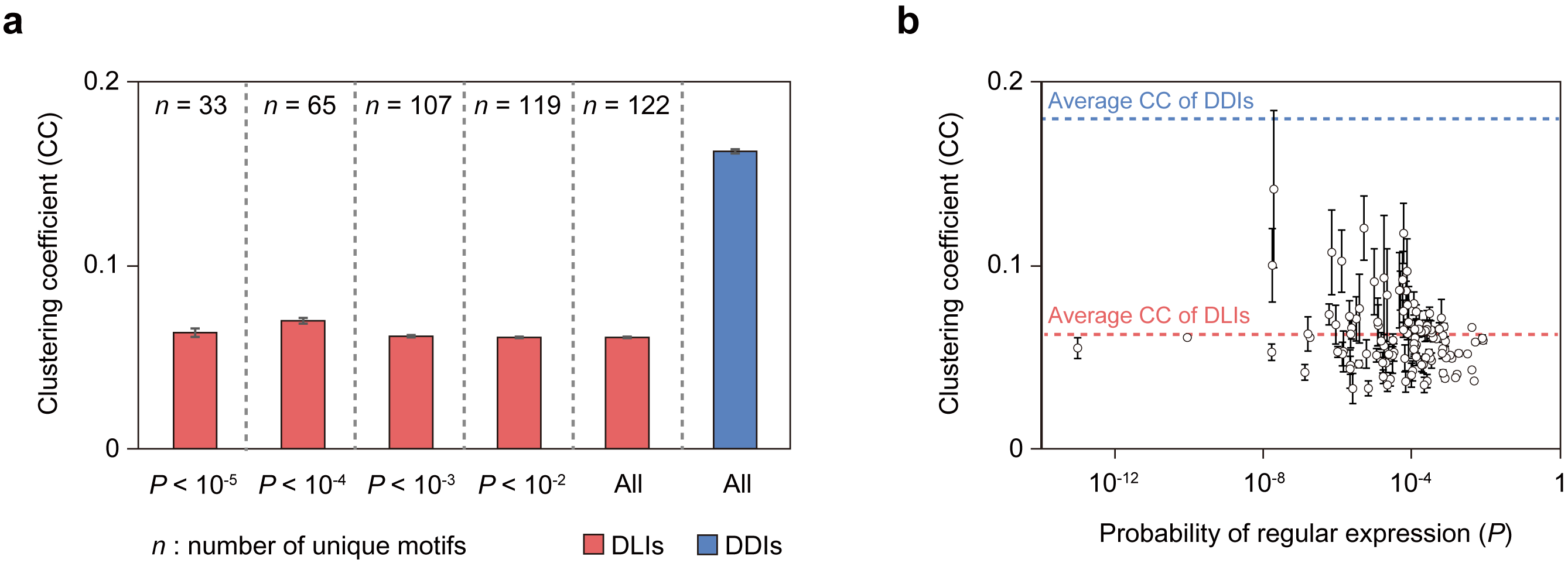

Supplement: Figure S2 — Clustering coefficients of DLIs according to the probability of motif regular expression. (a) Clustering coefficients of DLIs with varying probability cutoffs were compared with that of DDIs. (b) Each dot represents a motif with unique regular expression having its probability to be found by chance on x-axis, while average clustering coefficient of interactions on y-axis. The probability of motif regular expression was calculated as the product of the amino acid probability in each position of a motif [18]. (TIF) [file pcbi.1003881.s002.tif]

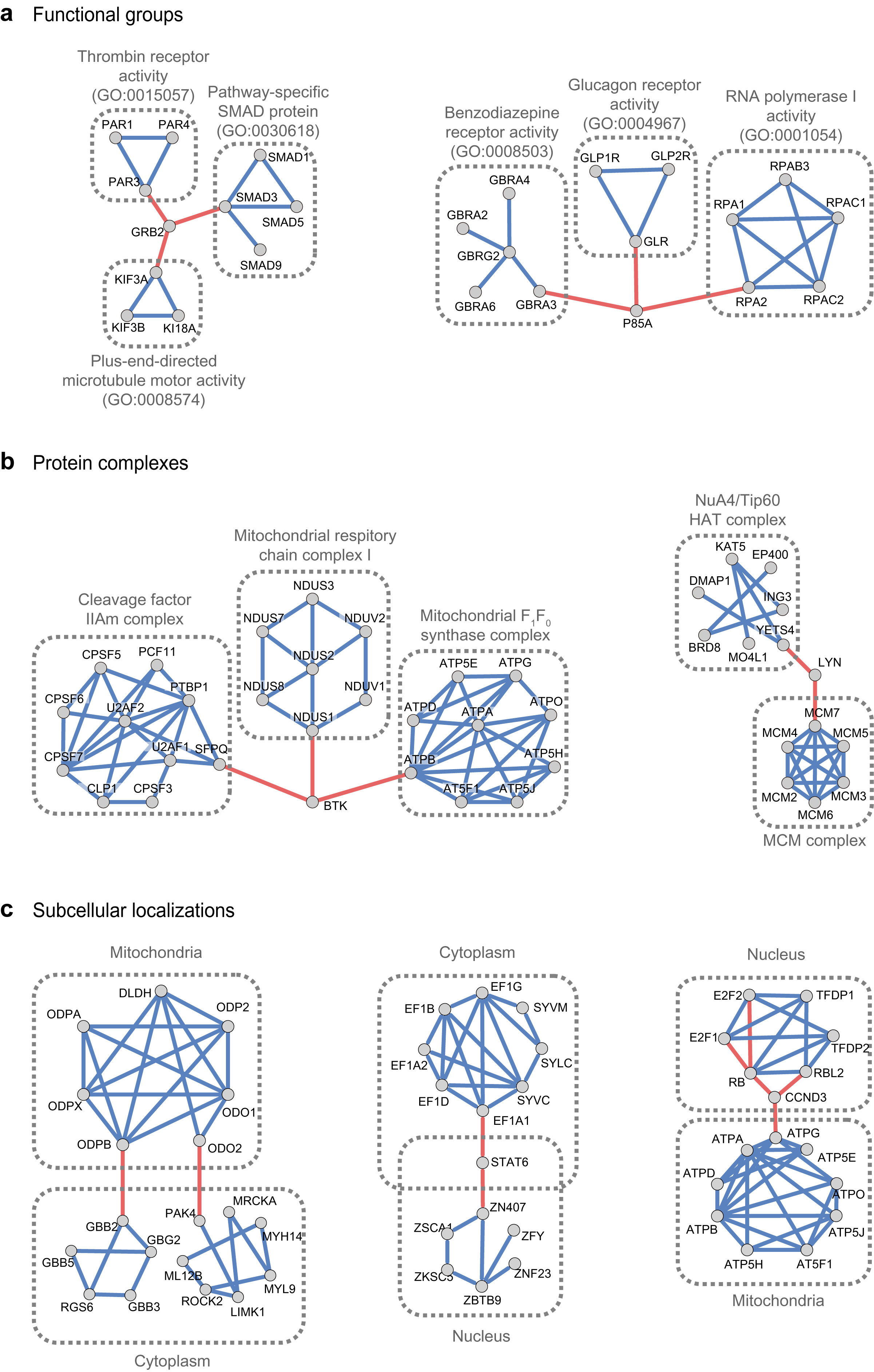

Supplement: Figure S3 — Examples of DLIs and DDIs in interactions between and within biological modules for (a) functional groups, (b) protein complexes, and (c) subcellular localizations. (TIF) [file pcbi.1003881.s003.tif]

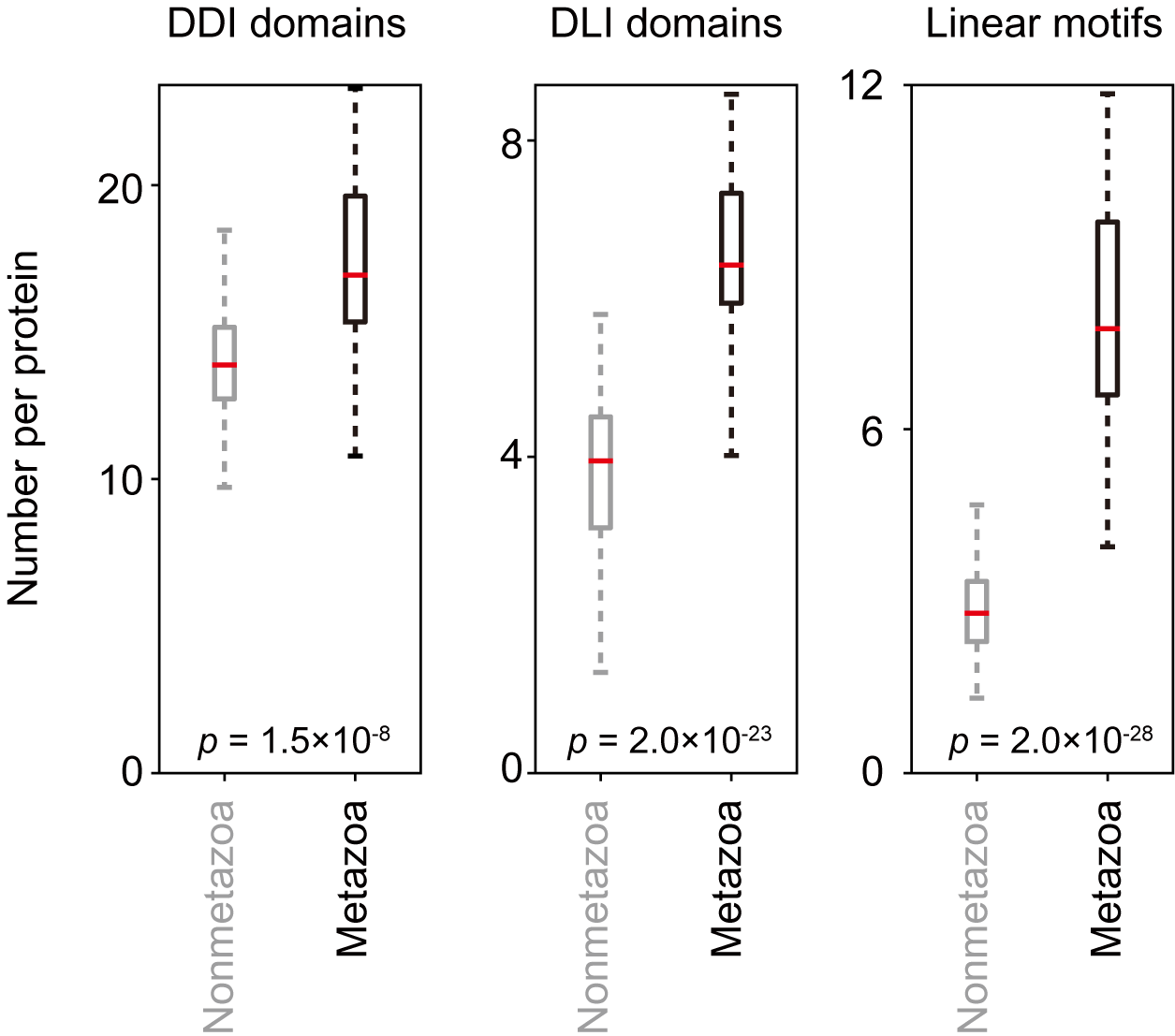

Supplement: Figure S4 — Number of domains and linear motifs mediating DDIs and DLIs in the PPI networks of eukaryotes. Average number per protein was calculated for each species and p-value was taken comparing 45 nonmetazoan and 53 metazoan species by t-test. (TIF) [file pcbi.1003881.s004.tif]

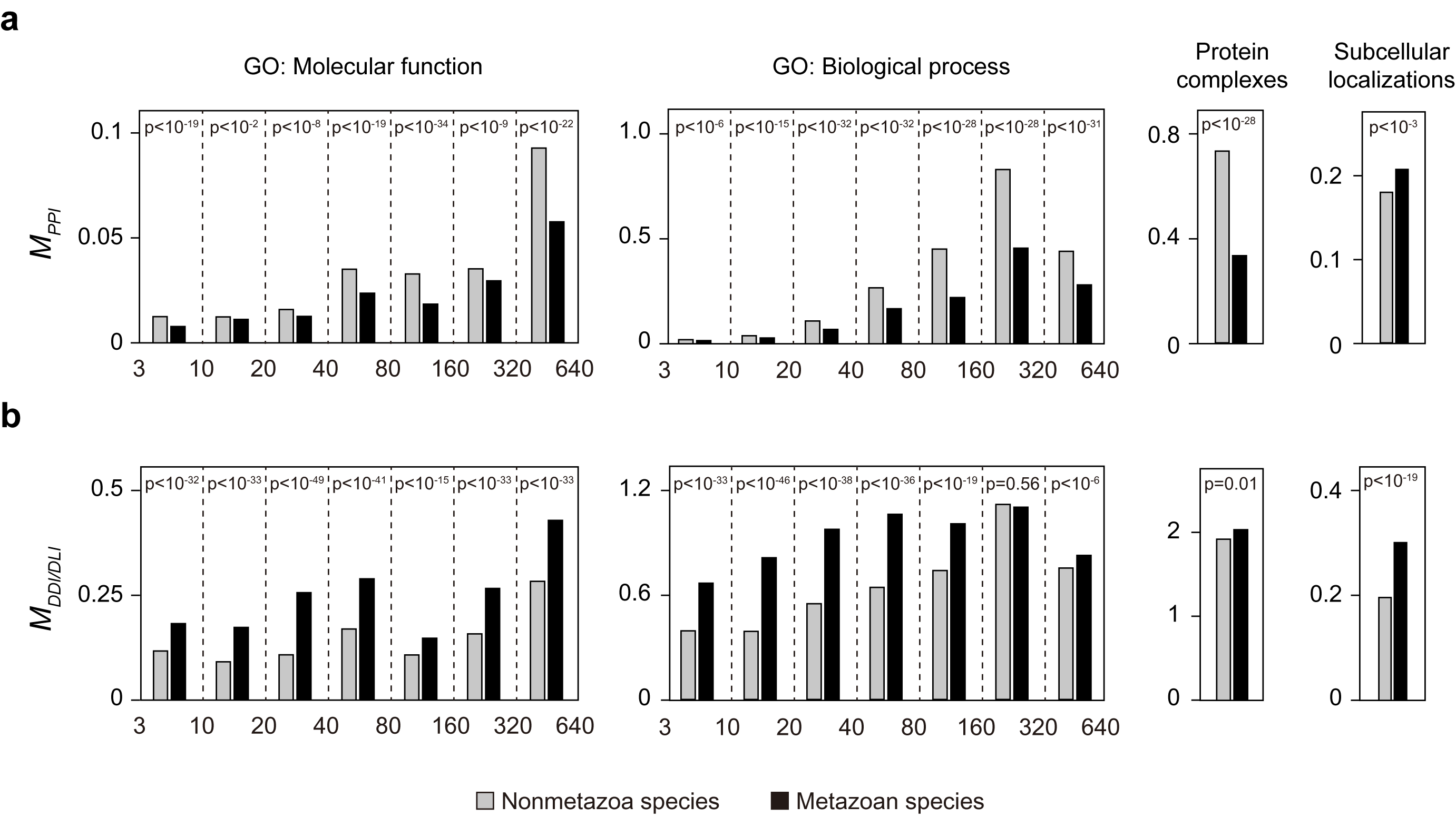

Supplement: Figure S5 — Enrichment of DLIs and DDIs in between and within-module interactions. Non-overlapping GO terms were used as functional groups. Module size represents the number of proteins in a given GO term. (TIF) [file pcbi.1003881.s005.tif]

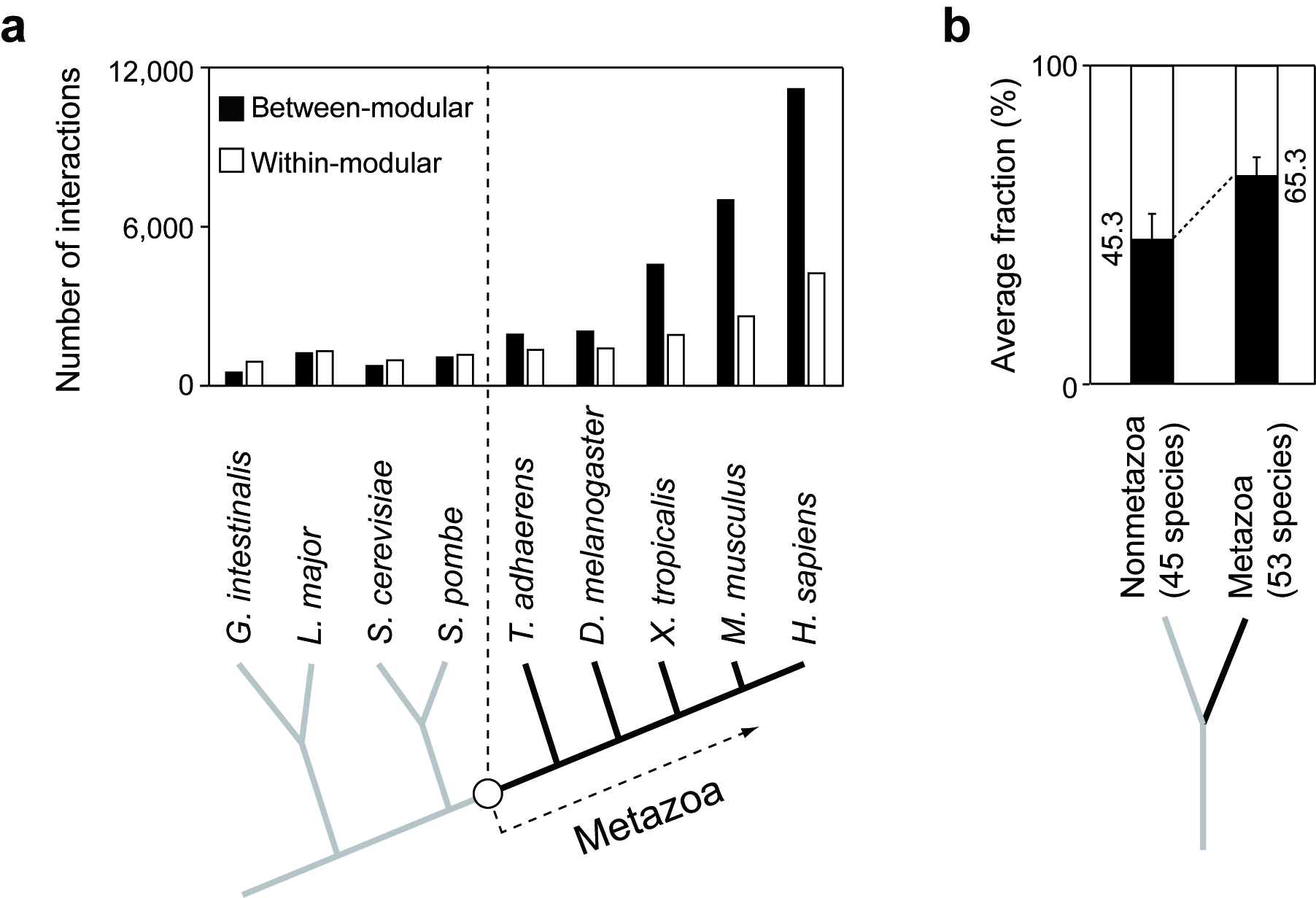

Supplement: Figure S6 — Enrichment homo-DDIs and DLIs in between and within-module interactions. (TIF) [file pcbi.1003881.s006.tif]

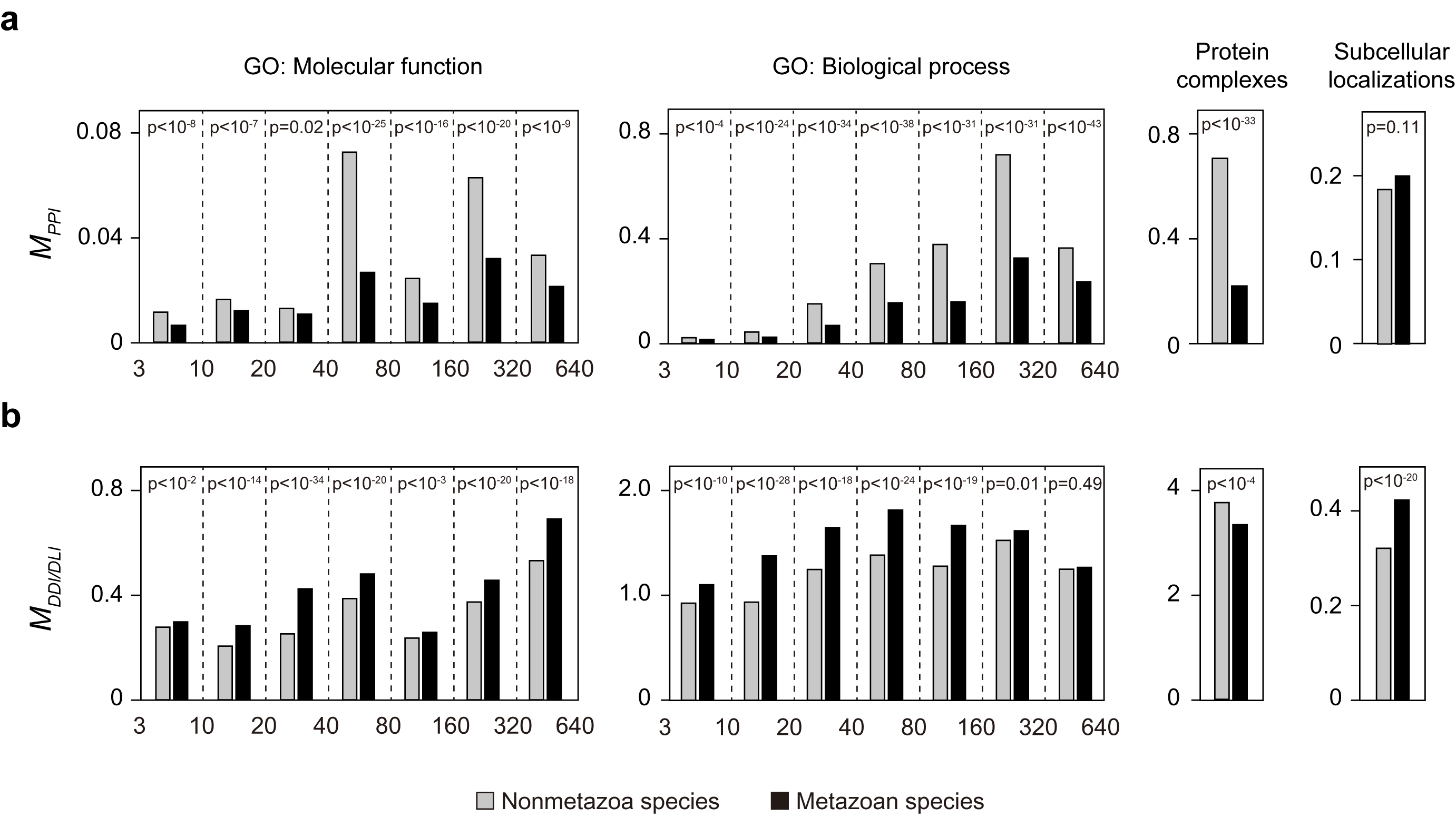

Supplement: Figure S7 — Enrichment hetero-DDIs and DLIs in between and within-module interactions. (TIF) [file pcbi.1003881.s007.tif]

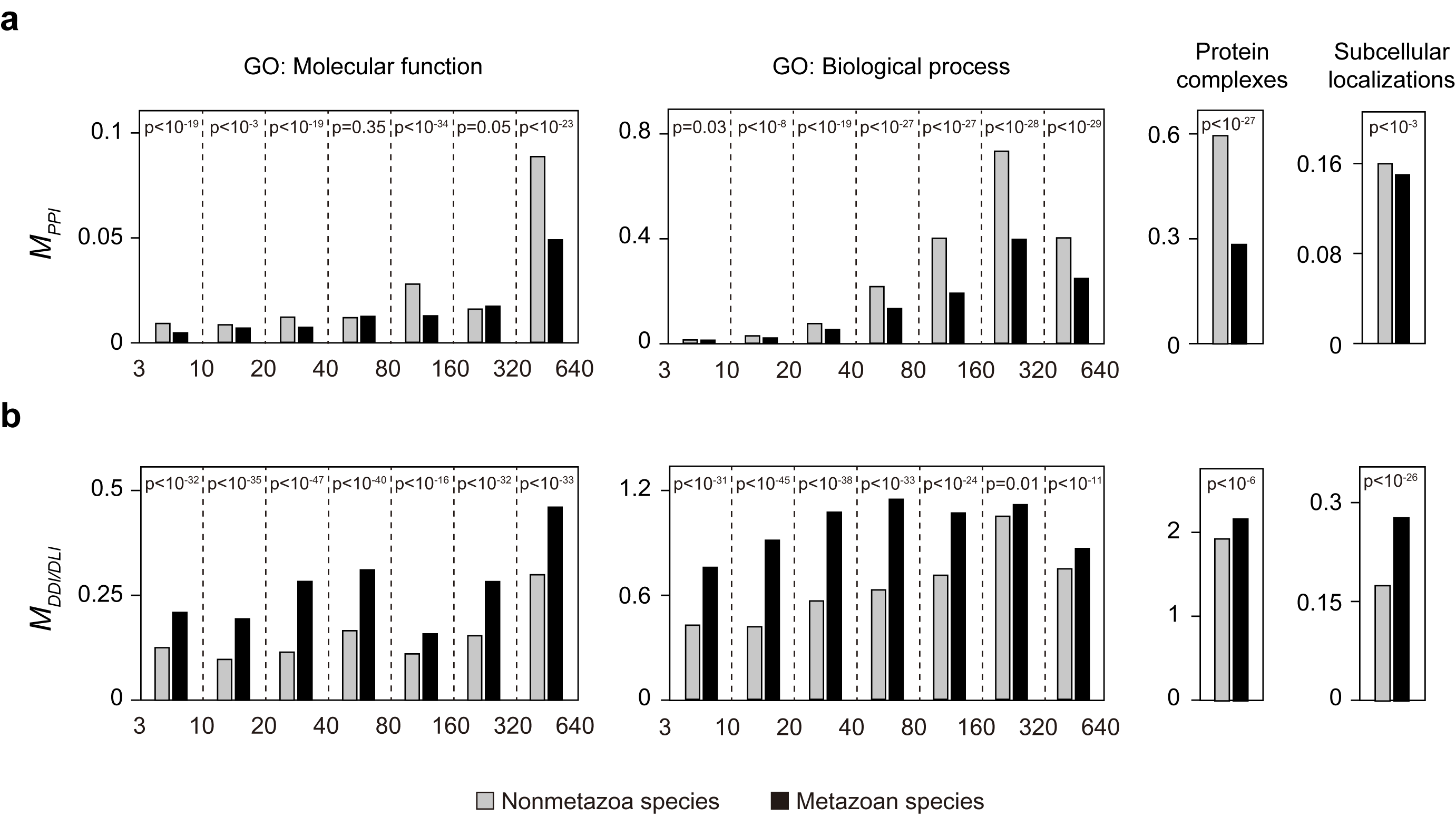

Supplement: Figure S8 — Increase in between-module interactions in metazoan PPI networks among protein complexes. (a) The number of between and within-module interactions in eukaryotic species among protein complexes. Values are shown for nine representative eukaryotic species. (b) Average proportion of between and within-module interactions in nonmetazoan and metazoan species. (TIF) [file pcbi.1003881.s008.tif]

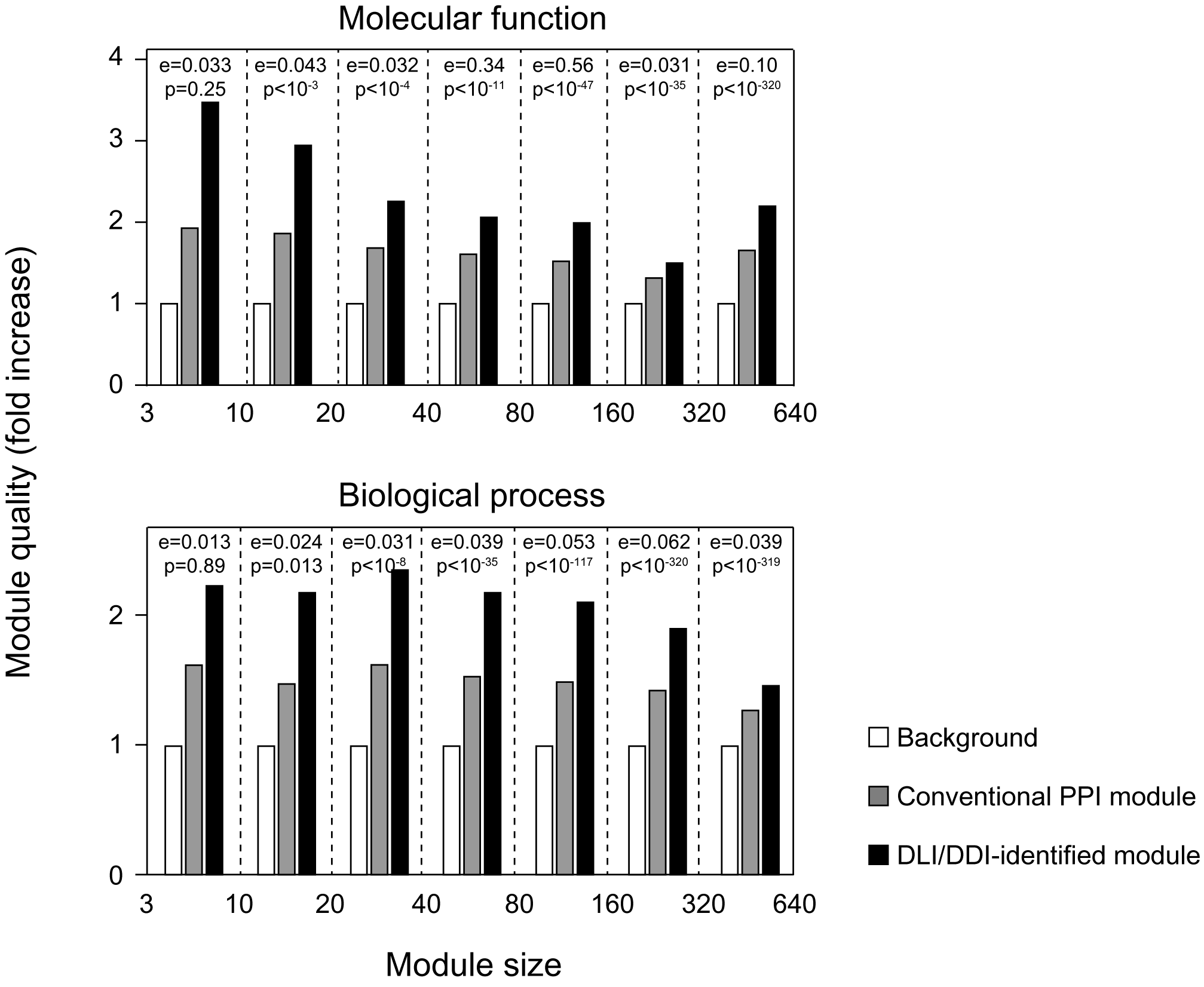

Supplement: Figure S9 — Comparison of module quality between DLI/DDI-identified modules and conventional PPI-identified modules for different functional groups. For both molecular function and biological process, the effect size, e, and the p-value are shown, stratified by module size. (TIF) [file pcbi.1003881.s009.tif]

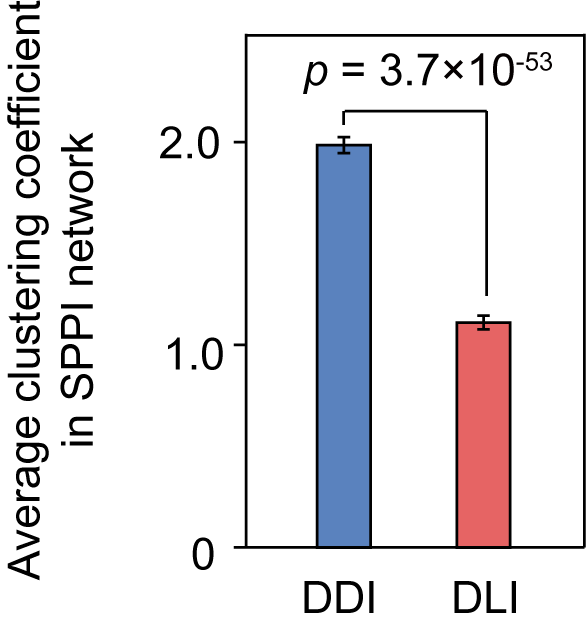

Supplement: Figure S10 — Edge clustering coefficient of DDIs and DLIs in SPPI network. (TIF) [file pcbi.1003881.s010.tif]

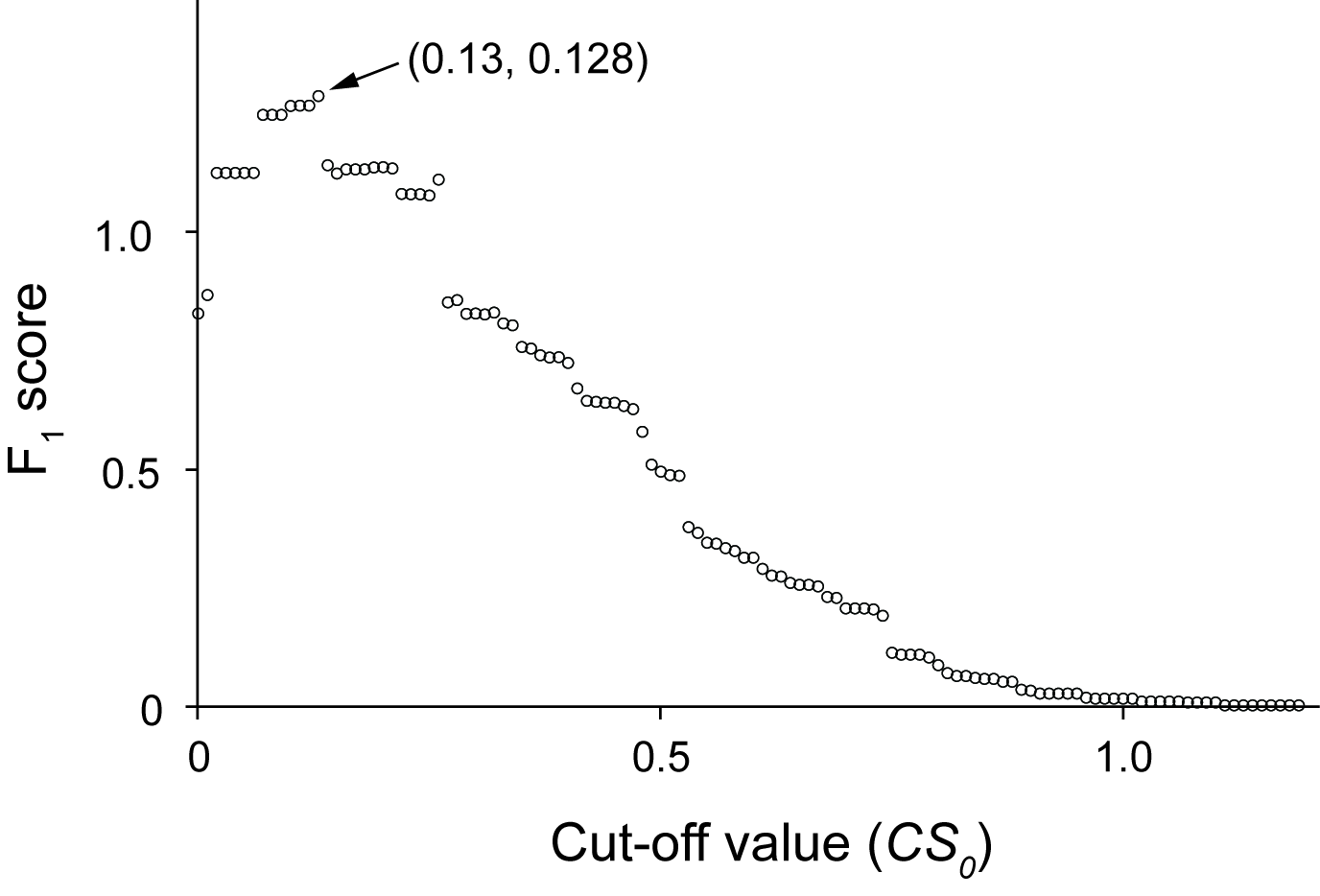

Supplement: Figure S11 — F1 score for the prediction of interacting domain-domain pairs in relation to the cut-off value, CS0. The F1 score was calculated for positive predictions, which were domain-domain pairs with a confidence score, CS, greater than the CS0. (TIF) [file pcbi.1003881.s011.tif]

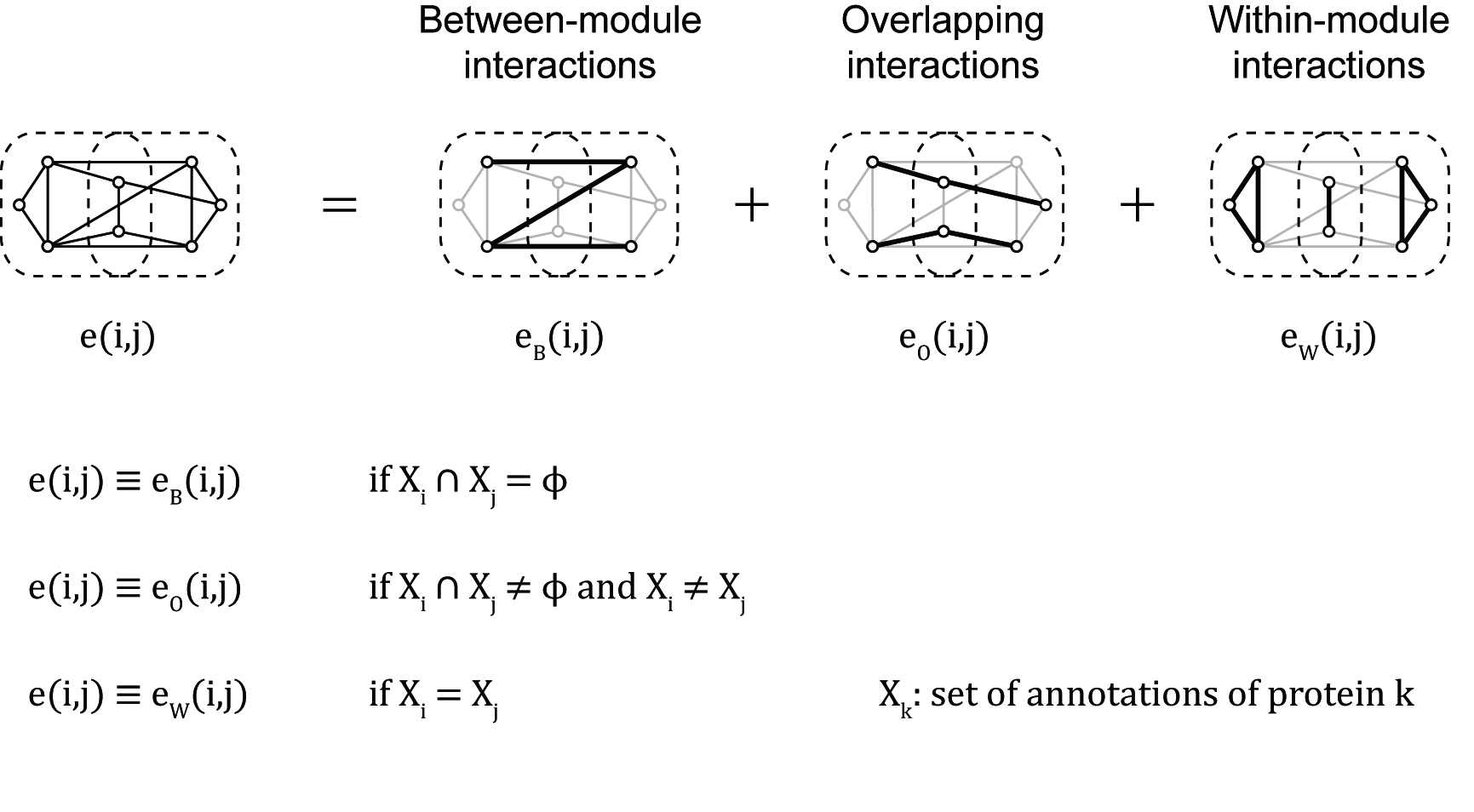

Supplement: Figure S12 — Schematic illustrating how PPIs were categorized as between or within-module interactions. (TIF) [file pcbi.1003881.s012.tif]
